# Chromosome numbers in antlions (Myrmeleontidae) and owlflies (Ascalaphidae) (Insecta, Neuroptera)

**DOI:** 10.3897/zookeys.538.6655

**Published:** 2015-11-19

**Authors:** Valentina G. Kuznetsova, Gadzhimurad N. Khabiev, Victor A. Krivokhatsky

**Affiliations:** 1 Zoological Institute, Russian Academy of Sciences, Universitetskaya nab. 1, 199034, St. Petersburg, Russia; 2 Saint Petersburg Scientific Center, Universitetskaya nab. 5, 199034, St. Petersburg, Russia; 3 Prikaspiyskiy Institute of Biological Resources, Dagestan Scientific Centre, Russian Academy of Sciences, ul. M. Gadzhieva 45, 367025 Makhachkala, Russia

**Keywords:** Male chromosome numbers, sex chromosomes, distant pairing of sex chromosomes, lacewings, Myrmeleontoidea

## Abstract

A short review of main cytogenetic features of insects belonging to the sister neuropteran families Myrmeleontidae (antlions) and Ascalaphidae (owlflies) is presented, with a particular focus on their chromosome numbers and sex chromosome systems. Diploid male chromosome numbers are listed for 37 species, 21 genera from 9 subfamilies of the antlions as well as for seven species and five genera of the owlfly subfamily Ascalaphinae. The list includes data on five species whose karyotypes were studied in the present work. It is shown here that antlions and owlflies share a simple sex chromosome system XY/XX; a similar range of chromosome numbers, 2n = 14-26 and 2n = 18-22 respectively; and a peculiar distant pairing of sex chromosomes in male meiosis. Usually the karyotype is particularly stable within a genus but there are some exceptions in both families (in the genera *Palpares* and *Libelloides* respectively). The Myrmeleontidae and Ascalaphidae differ in their modal chromosome numbers. Most antlions exhibit 2n = 14 and 16, and Palparinae are the only subfamily characterized by higher numbers, 2n = 22, 24, and 26. The higher numbers, 2n = 20 and 22, are also found in owlflies. Since the Palparinae represent a basal phylogenetic lineage of the Myrmeleontidae, it is hypothesized that higher chromosome numbers are ancestral for antlions and were inherited from the common ancestor of Myrmeleontidae + Ascalaphidae. They were preserved in the Palparinae (Myrmeleontidae), but changed via chromosomal fusions toward lower numbers in other subfamilies.

## Introduction

Within the holometabolous (= Endopterygota) insect order Neuroptera (lacewings) including a total of 17 or 18 currently recognized families (Aspöck et al. 2012), the Myrmeleontidae (antlions) comprise the most species-rich and most widespread family, with over 1500 valid extant species in 191 genera (Stange 2004). The closely related Ascalaphidae (owlflies) are a moderately speciose neuropteran family encompassing approximately 400 valid extant species assigned to about 65 genera, with wide distributional range in tropical and temperate areas of the world (Sekimoto and Yoshizawa 2007).

The Myrmeleontidae and Ascalaphidae belong to the superfamily Myrmeleontoidea (suborder Myrmeleontiformia), together with another four extant families, Nemopteridae, Crocidae, Psychopsidae, and Nymphidae. Despite the controversial hypotheses on the interfamilial phylogenetic relationships within this group, different phylogenetic analyses based on morphological and genetic data provide almost universal support for the monophyly of Myrmeleontoidea and the sister relationship between Myrmeleontidae and Ascalaphidae (Stange 1994, Aspöck 2002, Haring and Aspöck 2004, Winterton et al. 2010, Aspöck et al. 2012). However, molecular analyses are not always concordant with the monophyly of these families (Winterton et al. 2010).

Within Myrmeleontidae, the higher-level classification is controversial (reviewed in Mansell 1999), with several authors proposing various taxonomic divisions at the subfamily, tribe and subtribe levels (e.g. Banks 1899, 1927, New 1985a, b, c, Stange 1994, 2004, Krivokhatsky 2011). In his recent monography on the world fauna of Myrmeleontidae, Stange (2004) recognized three subfamilies, Stilbopteryginae, Palparinae, and Myrmeleontinae, with 14 tribes and 191 genera. Myrmeleontidae were further classified by Krivokhatsky (2011) who subdivided the family into 12 subfamilies (Palparinae, Pseudimarinae, Stilbopteryginae, Dimarinae, Echthromyrmicinae, Dendroleontinae, Nemoleontinae, Glenurinae, Myrmecaelurinae, Acanthaclisinae, Brachynemurinae, and Myrmeleontinae), with 23 tribes.

The Ascalaphidae are poorly-understood and taxonomically weakly-elaborated family. It was extensively revised only by van der Weele (1908) and now it comprises at least three subfamilies, Schizophthalminae (now Ascalaphinae), Holophthalminae (now Haplogleniinae), and Albardiinae, with a total of 15 tribes. Two-thirds of the species are placed in the first subfamily, and the remaining species (approximately 90) are placed in the second one, whereas the third subfamily contains only one species (van der Weele 1908, Sekimoto and Yoshizawa 2007). To date, no wide-ranging modern phylogenetic analyses of higher ascalaphid relationships have been published (Fischer et al. 2006).

Mansell (1999: p. 3) pointed out that the antlions, “apart from their obvious biological significance, are ideal subjects for the study of insect behavior, physiology, biogeography and evolution, and consequently a group urgently warrants study and conservation”. Although chromosomal investigations have a long history in systematics and evolutionary biology (White 1973, King 1993), and a large body of data has been accumulated for insects (e.g., butterflies: Lukhtanov 2014; beetles: Angus et al. 2013, Blackmon and Demuth 2014, 2015; true bugs: Papeschi and Bressa 2006, Kuznetsova et al. 2011; aphids: Gavrilov-Zimin et al. 2015; coccids: Gavrilov 2007; cicadas: Kuznetsova and Aguin-Pombo 2015; grasshoppers: Warchałowska-Śliwa et al. 2005, parasitic wasps: Gokhman 2009), both antlions and owlflies were largely ignored in this respect. Our present knowledge of their karyotypes is scarce and fragmentary, being completely confined to the number of chromosomes and, additionally, to the meiotic behavior of the sex chromosomes that is of a very peculiar type in many neuropteran groups (Naville and de Beaumont 1932, 1933, Hughes-Schrader 1969, 1975a, b, 1979, Nokkala 1986) including the Myrmeleontidae (Naville and de Beaumont 1932, 1933, Hughes-Schrader 1983). In the Myrmeleontidae and Ascalaphidae, chromosomal studies were initiated in the 1930s with the pioneering works of Oguma and Asana (1932), Naville and de Beaumont (1932, 1933, 1936), Ikeda and Kichijo (1935), Asana and Kichijo (1936), and Katayama (1939). Since that time only scarce chromosome studies were performed on the Myrmeleontidae (Hirai 1955a, b, Highes-Schrader 1983, Klok and Chown 1993) while no further work on the Ascalaphidae appeared except for the re-investigation of *Ascalohybris subjacens* (Walker, 1853) karyotype (Hirai 1955a, b: as *Hybris* Lefèbvre, 1842) earlier studied by Katayama (1939: as *Hybris*).

Thus, cytogenetic studies on the families Myrmeleontidae and Ascalaphidae virtually ceased a few decades ago. The latest checklist of chromosome numbers in antlions published by Klok and Chown (1993) suffers from many shortcomings including imperfect references, erroneous identifications, outdated species names and synonymy. In order to fill this gap, an updated and comprehensive checklist of chromosome numbers of antlions and owlflies is provided here by integrating the published data together with our latest unpublished results.

## Material and methods

### Insects

Four antlion species (only males), namely *Palpares libelluloides*, *Distoleon tetragrammicus*, *Macronemurus bilineatus*, *Myrmecaelurus trigrammus*, and male owlfly *Bubopsis hamatus*, were used in the present study. The specimens were collected from May to October 2013 in the Republic of Dagestan (North-East Caucasus, Russia). The material was collected by G. Khabiev. Collection sites, sampling dates, and the numbers of studied males are given in Table 1. In the field, adult individuals were fixed in a solution of 96% alcohol and glacial acetic acid (3:1) and then stored at 4 °C until required.

**Table 1.** Material used.

Taxon	Sampling locality and date of collection	No. of studied males
**Myrmeleontidae**		
**Palparinae**		
*Palpares libelluloides* (Linnaeus, 1764)	Russia, Dagestan, near Makhachkala 43°00'00"N, 47°13'33"E; V.2013	2
**Nemoleontinae**		
*Distoleon tetragrammicus* (Fabricius, 1798)	Russia, Dagestan, near Makhachkala 43°00'29"N, 47°14'51"E VII.2013	1
*Macronemurus bilineatus* Brauer, 1868	Russia, Dagestan, near Makhachkala 42°59'58"N 47°13'30"E; VI.2013	7
**Myrmecaelurinae**		
*Myrmecaelurus trigrammus* (Pallas, 1771)	Russia, Dagestan, near Makhachkala 43°01'26"N, 47°15'12"E; 42°57'19"N, 47°28'51"E; 42°58'07.2"N, 47°20'03"E; VI-VII.2013	23
**Ascalaphidae**		
*Bubopsis hamatus* (Klug in Ehrenberg, 1834)	Dagestan, Gumbetovsky district, near Chirkata village; 42°47'53"N, 46°41'14"E; VII.2013	2

### Chromosome preparation

Air-dried preparations were made by macerating testicular follicles in a drop of 45% acetic acid on a glass microscope slide and squashing under a cover slip. The preparations were frozen using dry ice, the cover slips were removed with a razor blade, and the preparations were dehydrated in fresh fixative (3:1) for 20 min and air dried. Slides were first examined under a phase-contrast microscope to check for the availability of meiotic divisions and quality of chromosome spreads. Counts were based on samples of one to 23 individuals. The preparations and remains of the specimens are stored at the Department of Karyosystematics, Zoological Institute, RAS.

### Chromosome staining

Meiotic chromosomes were stained using the Feulgen-Giemsa method developed by Grozeva and Nokkala (1996).

### Microscopy and imaging

Chromosome preparations were analyzed under a Leica DM 4000B microscope with a 100x objective. Images were taken with a Leica DFC 345 FX camera using Leica Application Suite 3.7 software with an Image Overlay module.

## Results

**Figures 1–5.**
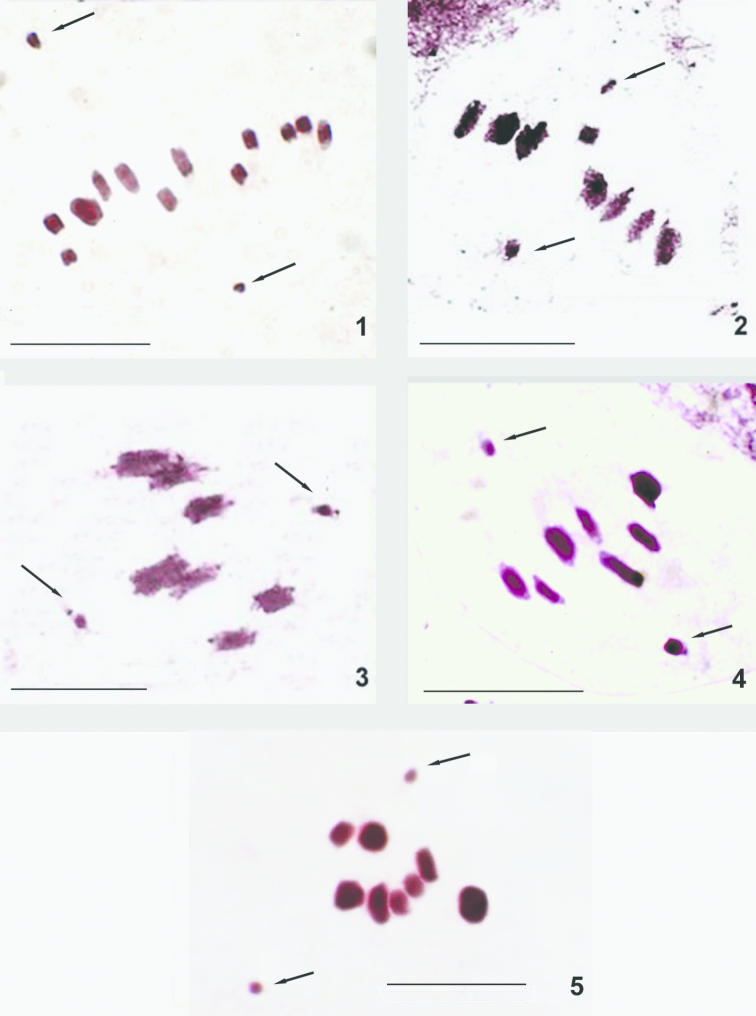
Meiotic (MI) karyotypes of antlions (**1–4**) and owlflies (**5**). **1** *Palpares libelluloides*, n = 12AA+XY (2n = 26, XY) **2** *Distoleon tetragrammicus*, n = 8AA+XY (2n = 18, XY) **3** *Myrmecaelurus trigrammus*, n = 7AA+XY (2n = 16, XY) **4** *Macronemurus bilineatus*, n = 7AA+XY (2n = 16, XY), **5** *Bubopsis hamatus*, n = 8AA+XY (2n = 18, XY). Arrows point to X and Y sex chromosomes. Scale bars = 10 µm

Only meiotic divisions in adult males were available for analysis during the present study. In five examined species belonging to the families Myrmeleontidae (four species) and Ascalaphidae (one species) (Table 1), as many as three different chromosome numbers were found. Males of *Palpares libelluloides* showed 12 autosomal bivalents and univalent X and Y chromosomes at spermatocyte metaphases I (MI) suggesting the diploid karyotype formula of this species is 2n = 26(24A + XY). Unfortunately, our method proved to be inappropriate for effective and reliable detection of the centromere positions in chromosomes and, hence, analysis of their morphology. Nonetheless, most autosomes were suggested to be one-armed, with at least one clear exception of a large pair of bi-armed submetacentric chromosomes (Fig. 1). Males of *Macronemurus bilineatus* and *Myrmecaelurus trigrammus* showed 7 autosomal bivalents and univalent X and Y chromosomes at spermatocyte MI suggesting the diploid karyotype formula is 2n = 16(14A + XY). Males of *Distoleon tetragrammicus* and *Bubopsis hamatus* showed 8 autosomal bivalents and univalent X and Y chromosomes at spermatocyte MI suggesting the diploid karyotype formula is 2n = 18(16A + XY). In the four low-numbered species, the chromosomes seemed to be essentially bi-armed (Figs 2–5).

A peculiar feature of all the species was that at metaphase I, the univalent X and Y chromosomes were disposed on the opposite sides of the division spindle whereas autosomal bivalents showed a typical metaphase location on the equator of the nucleus (Figs 1–5). In each species, the behavior of sex chromosomes was traced in the meiotic nuclei throughout all stages and these data will be presented elsewhere.

The new findings and references to previous reports of chromosome numbers in Myrmeleontidae and Ascalaphidae are given in Table 2. The subfamilial and tribal classification of the Myrmeleontidae used in this paper follows Krivokhatsky (2011) and that of the Ascalaphidae follows van der Weele (1908).

**Table 2.** Data on karyotypes in the Myrmeleontidae and the Ascalaphidae (Neuroptera: Myrmeleontoidea).

No	Taxon	2n (karyotype formula) ♂	Sampling locality	Reference
	**Family Myrmeleontidae Latreille, 1802**			
	**Subfamily Palparinae Banks, 1911**			
1	*Indopalpares pardus* (Rambur, 1842)	24(22+XY)	East India: Ahmedabad	Oguma and Asana 1932 (as *Palpares* sp.)^1^
2	*Palpares libelluloides* (Linnaeus, 1764)	26(24+XY) 26(24+XY)	Switzerland: Ge♀neve, France: Banyuls-sur-Mer Russia: Dagestan	Naville and De Beaumont 1936 Present data
3	*Palpares sobrinus* Péringuey, 1911	22(20+XY)	South Africa: Transvaal	Klok and Chown 1993
	**Subfamily Pseudimarinae Markl, 1954**			
	**Tribe Palparidiini Markl, 1954**			
4	*Palparidius concinnus* Péringuey, 1910	18(16+XY)	South Africa: Transvaal	Klok and Chown 1993
	**Subfamily Dendroleontinae Banks, 1899**			
	**Tribe Dendroleontini Banks, 1899**			
5	*Epacanthaclisis moiwanus* (Okamoto, 1906)	16(14+XX) (♀)	Japan	Hirai 1955a, b
6	*Dendroleon jezoensis* Okamoto, 1910	16(14+XY)	Japan	Hirai 1955a, b
	**Subfamily Nemoleontinae Banks, 1911**			
	**Tribe Distoleontini Tillyard, 1916**			
7	*Distoleon tetragrammicus* (Fabricius, 1798)	18(16+XY)	Russia: Dagestan	Present data
	**Tribe Neuroleontini Banks, 1911**			
8	*Neuroleon* sp.^2^	16(14+XY)	Western India: Bombay [Mumbai]	Asana and Kichijo 1936
	**Tribe Macronemurini Esben-Petersen, 1919**			
9	*Macronemurus appendiculatus* (Latreille, 1807)	16(14+XY)	France: Banyuls-sur-Mer	Naville and De Beaumont 1933
10	*Macronemurus bilineatus* Brauer, 1868	16(14+XY)	Russia: Dagestan	Present data
11	*Macronemurus* sp.	16(14+XY)	Western India: Bombay [Mumbai]	Asana and Kichijo 1936 (as *Macronemurus* sp.?)
	**Tribe Creoleontini Markl, 1954**			
12	*Creoleon lugdunensis* (Villers, 1789)	18(16+XY)	France: Banyuls-sur-Mer	Naville and De Beaumont 1936 (as *Creagris plumbea* Navás, 1928)^3^
	**Subfamily Glenurinae Banks, 1927**			
	**Tribe Glenurini Banks, 1927**			
13	*Euptilon arizonensis* (Banks, 1935)	16(14+XY)	USA	Hughes-Schrader 1983 (as *Psammoleon arizonensis* Banks, 1935)
14	*Paraglenurus japonicus* (MacLachlan, 1867)	16(14+XY)	Japan	Hirai 1955a, b (as *Glenuroides japonicus* MacLachlan, 1867)
	**Subfamily Myrmeleontinae Latreille, 1802**			
	**Tribe Myrmeleontini Latreille, 1802**			
15	*Baliga micans* (McLachlan, 1875)	14(12+XY)	Japan	Hirai 1955a, b (as *Hagenomyia micans* McLachlan, 1875)
16	*Baliga sagax* (Walker, 1853)	14(12+XY)	Western India: Bombay [Mumbai]	Asana and Kichijo 1936 (as *Myrmeleon* sp., probably *Myrmeleon sagax* Walker, 1853)
17	*Euroleon nostras* (Fourcroy, 1785)	14(12+XY) 14(12+XX) (♀)	Switzerland, Geneva	Naville and De Beaumont 1932, 1933 (as *Myrmeleon europaeus* McLachlan, 1873)
18	*Myrmeleon alcestris* Banks, 1911	14(12+XY)	South Africa: Transvaal	Klok and Chown 1993
19	*Myrmeleon californicus* Banks, 1943	14(12+XY)	USA	Hughes-Schrader 1983
20	*Myrmeleon exitialis* Walker, 1853	14(12+XY)	USA	Hughes-Schrader 1983
21	*Myrmeleon formicarius* Linnaeus, 1767^2^	14(12+XY) 14(12+XY)	Western India: Bombay [Mumbai] Japan	Ikeda and Kichijo 1935, Hirai 1955a, b
22	*Myrmeleon hyalinus* Olivier, 1811	14(12+XY)	France: Corse	Naville and De Beaumont 1936 (as *Morter hyalinus* (Olivier, 1811))
23	*Myrmeleon immaculatus* DeGeer, 1773	14(12+XY)	USA	Hughes-Schrader 1983
24	*Myrmeleon mexicanus* Banks, 1903	14(12+XY)	USA	Hughes-Schrader 1983
25	*Myrmeleon obscurus* Rambur, 1842	14(12+XY)	South Africa: Transvaal	Klok and Chown 1993
	**Subfamily Brachynemurinae Banks, 1927**			
	**Tribe Brachynemurini Banks, 1927**			
26	*Brachynemurus hubbardi* Currie, 1898	14(12+XY)	USA	Hughes-Schrader 1983
27	*Brachynemurus mexicanus* Banks, 1895	14(12+XY)	USA	Hughes-Schrader 1983
28	*Clathroneuria coquilletti* (Currie, 1898)	14(12+XY)	USA	Hughes-Schrader 1983 (as *Brachynemurus coquilletti* Currie, 1898)
29	*Clathroneuria schwarzi* (Currie, 1903)	14(12+XY)	USA	Hughes-Schrader, 1983 (as *Brachynemurus schwarzi* Currie, 1903)
30	*Scotoleon dissimilis* (Banks, 1903)	16(14+XY)	USA	Hughes-Schrader 1983 (as *Brachynemurus dissimilis* Banks, 1903)
31	*Scotoleon niger* (Currie, 1898)	16(14+XY)	USA	Hughes-Schrader 1983 (as *Brachynemurus niger* Currie, 1898)
32	*Scotoleon nigrilabris* (Hagen, 1888)	16(14+XY)	USA	Hughes-Schrader 1983 (as *Brachynemurus nigrilabris* Hagen, 1888)
	**Subfamily Myrmecaelurinae Esben-Petersen, 1919**			
	**Tribe Myrmecaelurini Esben-Petersen, 1919**			
33	*Myrmecaelurus* sp.^2^	14(12+XY)	Western India: Bombay [Mumbai]	Asana and Kichijo 1936 (as *Myrmecaelurus* sp, probably *Myrmecaelurus acerbus* (Walker, 1853))
34	*Myrmecaelurus trigrammus* (Pallas, 1771)	16(14+XY)	Russia: Dagestan	Present data
	**Subfamily Acanthaclisinae Navás, 1912**			
35	*Synclisis japonica* (McLachlan, 1875)	14(12+XY) 14(12+XY)	Western India: Bombay [Mumbai] Japan	Ikeda and Kichijo 1935, Hirai 1955a, b (as *Acanthaclisis japonica* Hagen, 1866)
36	*Centroclisis brachygaster* (Rambur, 1842)	14(12+XY)	South Africa: Transvaal	Klok and Chown 1993
37	*Vella fallax* (Rambur, 1842)	14(12+XY)	USA	Hughes-Schrader 1983
	**Family Ascalaphidae Rambur, 1842**			
	**Subfamily Ascalaphinae Rambur, 1842**			
	**Tribe Hybrisini Lefèbvre, 1842**			
38	*Ascalohybris subjacens* (Walker, 1853)	22(20+XY) 22(20+XX) (♀)	Japan Japan	Katayama 1939 (as *Hybris subjacens* (Walker, 1853)), Hirai 1955a, b (as *Hybris subjacens*)
39	*G1yptobasis dentifera* (Westwood, 1847)	22(20+XY)	Western India: Bombay [Mumbai]	Asana and Kichijo 1936
	**Tribe Ascalaphini Rambur, 1842**			
40	*Libelloides corsicus* Rambur, 1842)	20	France: Corse	Naville and De Beaumont 1936 (as *Ascalaphus ictericus corsicus* Rambur, 1842)
41	*Libelloides coccajus* (Denis & Schiffermüller, 1775)	22(20+XY) 22(20+XX) (♀)	Switzerland: Geneva, Valais	Naville and De Beaumont 1933, 1936 (as *Ascalaphus libelluloides* Schäffer, 1763)
42	*Libelloides longicornis* (Linnaeus, 1764)	22(20+XY)	Switzerland: Valais	Naville and De Beaumont 1936 (as *Ascalaphus longicornis* (Linnaeus, 1764))
	**Tribe Encyoposini McLachlan, 1871**			
43	*Bubopsis hamatus* (Klug in Ehrenberg, 1834)	18(16+XY)	Russia: Dagestan	Present data
44	*Ogcogaster segmentator* (Westwood, 1847)	22(20+XY)	Western India: Bombay [Mumbai]	Asana and Kichijo 1936

1 Later described as Palpares pardus asanai Kuwayama, 1933 (Oguma and Asana 1932, Kuwayama 1933)

2 Presence of these taxa in Bombay [Mumbai] is doubtful

3 Wrong identifications: all records of Creoleon plumbeus from West Europe actually belong to Creoleon lugdunensis (Hölzel 1976, Krivokhatsky 2011)

## Discussion

### Chromosome numbers

In the Myrmeleontidae, with the original data presented here, karyotype data have been made available for 37 species and 21 genera in 9 out of 12 subfamilies accepted by Krivokhatsky (2011). Having regard to 1500 valid species and 191 valid genera in this family (Stange 2004), the proportion of the studied species and genera is approximately 2.5% and 11% respectively. The karyotypes (chromosome numbers and sex chromosome systems) are presently known for the subfamilies Palparinae (3 species/2 genera), Pseudimarinae (1/1), Dendroleontinae (2/2), Nemoleontinae (6/4), Glenurinae (2/2), Myrmeleontinae (11/3), Brachynemurinae (7/3), Myrmecaelurinae (2/1), and Acanthaclisinae (3/3). The family demonstrates a relatively high diversity of karyotypes, with diploid chromosome numbers (2n) of 37 studied species ranging from 14 to 26 including four intermediate counts, i.e. 16, 18, 22 and 24. The highest numbers, 26, 24 and 22, occur only in the subfamily Palparinae, in three species of the genera *Palpares* Rambur, 1842 and *Indopalpares* Insom & Carfi, 1988. Other numbers, 2n = 14, 16 and 18, are encountered in the remaining subfamilies. In the Pseudimarinae, the only studied species, *Palparidius concinnus*, exhibits the next highest number found in antlions, i.e. 2n = 18. In the Nemoleontinae, with the exception of *Distoleon tetragrammicus* and *Creoleon lugdunensis* displaying 2n = 18, all studied species, including three *Macronemurus* Costa, 1855 species, have karyotypes with 2n = 16. The latter value is also found in all studied Dendroleontinae and Glenurinae. The subfamilies Brachynemurinae, Myrmecaelurinae and Acanthaclisinae include species both with 2n = 16 and 2n = 14, whereas Myrmeleontinae show 2n = 14 in all the studied species. It is noteworthy that, with the exception of *Palpares* (but see below), all these genera do not show interspecific variation in the chromosome number. This is especially remarkable for those genera where several species have been studied, e.g. *Macronemurus* (Nemoleontinae) and *Scotoleon* Banks, 1913 (Brachynemurinae). In each of these genera, three studied species share 2n = 16. Moreover, in *Myrmeleon* Linnaeus, 1767 (Myrmeleontinae) all eight studied species have 2n = 14. It is noteworthy that closely related genera, *Baliga* Navás, 1912 and *Euroleon* Esben-Petersen, 1918 in the Myrmeleontini, show the same karyotype with 2n = 14. It is unclear at present whether the chromosome number varies within the genus *Palpares*. The highest chromosome number, 2n = 26, is found in *Palpares libelluloides*, the type species of the genus. *Palpares pardus asanai* Kuwayama, 1933 with 2n = 24 (Oguma and Asana 1932) is treated here as a member of *Indopalpares*. Additionally, there is a possibility that *Palpares sobrinus* with 2n = 22 (Klok and Chown 1993) represents in fact *Pseudopalpares sparsus* (McLachlan, 1867). Although few members of the Palparinae are studied at present, karyotypic differences between the genera of this subfamily probably occur.

Compared to the Myrmeleontidae, karyotypes of the Ascalaphidae are less studied. The chromosome numbers are currently known in only seven owlfly species from the genera *Ascalohybris* Sziraki, 1998, *Ogcogaster* Westwood, 1847, *Libelloides* Schaeffer, 1766, *Bubopsis* McLachlan, 1898, and *Glyptobasis* McLachlan, 1871, all presently classified within the subfamily Ascalaphinae. The species studied show relatively high chromosome numbers, i.e. 2n = 18 in *Bubopsis hamatus*, 20 in *Libelloides corsicus*, and 22 in all the remaining species, including two other studied members of the genus *Libelloides*.

Although Myrmeleontidae and Ascalaphidae show a similar range of chromosome numbers (2n = 14 - 26 in the former and 18 - 22 in the latter), these families differ in the modal numbers. Of 37 species studied in the Myrmeleontidae, 19 species display 2n = 14, and 12 species have 2n = 16. On the other hand, five of seven species studied in the Ascalaphidae display 2n = 22. Other chromosome numbers occur only occasionally within the families except for high numbers characteristic of the antlion subfamily Palparinae.

In different eukaryotic organisms, evolutionary changes in the chromosome number happen via polyploidy, aneuploidy or fusion/fission events. In animals polyploidy is known to be rare, whereas chromosomal fusions and fissions are common. As stated above, most Myrmeleontidae possess lower chromosome numbers, 2n = 14 and 2n = 16, which are encountered in all subfamilies, with the only exception of the Palparinae. The latter is the only subfamily characterized by higher numbers, 2n = 26, 24, and 22, and the higher number, 2n = 18, is also found in the only studied species of the related subfamily Pseudimarinae. The higher numbers, 2n = 22, 20 and 18, are also characteristic of the sister family Ascalaphidae. Since Palparinae represent a basal phylogenetic lineage of the Myrmeleontidae (Krivokhatsky 2011), it is hypothesized that higher chromosome numbers are ancestral for antlions. Most likely, the higher chromosome numbers were inherited from the common ancestor of Myrmeleontidae + Ascalaphidae. It was preserved in the subfamily Palparinae (Myrmeleontidae) but changed via chromosomal fusions toward lower numbers, 2n = 18, 16 and 14, in other subfamilies.

Knowledge of the chromosome morphology in the low-numbered and high-numbered chromosome complements would help in understanding the karyotype evolution in the Myrmeleontidae and Ascalaphidae and testing the above hypothesis. Unfortunately, despite several efforts to identify chromosomal morphology within particular karyotypes (e.g. Asana and Kichijo 1936, Hughes-Schrader 1983, present study), this important question remains unresolved. Special staining methods, e.g. C-banding, are therefore needed to identify the centromeric position in the chromosomes and thus their morphology. However, these techniques have never been used in neuropteran cytogenetics, and therefore this is the most serious objective in the chromosome research of antlions and owlflies.

### Sex chromosome system

All Myrmeleontidae and Ascalaphidae species, including those studied here, exhibit a simple sex chromosome system XY/XX, which is characteristic of the whole order Neuroptera (White 1973, Blackman 1995). Both antlions and owlflies demonstrate a very peculiar behavior of sex chromosomes in males (Naville and de Beaumont 1933, 1936, Asana and Kichijo 1936, Hughes-Schrader 1983, Klok and Chown 1993, present paper). In spermatocyte meiosis of those insects, sex chromosomes take up positions at opposite halves of the meiotic spindle at metaphase I before segregating into the daughter spermatocytes. It means that the X and Y chromosomes get segregated to opposite poles of the spindle long before the autosomal half-bivalents disjoin at anaphase I and move to the poles. The same pattern, the so-called “distance pairing” of sex chromosomes first discovered by Naville and de Beaumont (1933) in antlions, is known to be characteristic of the related neuropteran families Chrysopidae, Mantispidae, Sisyridae, Osmylidae, and Hemerobiidae (Naville and de Beaumont 1936, Hughes-Schrader 1969, 1975b, 1980, Nokkala 1986) and probably of the order Neuroptera in general. The biological role of this unusual behavior of sex chromosomes is unclear. In any case, this mechanism observed in brown lacewings (Hemerobiidae) showed no significance for the regular segregation of the sex chromosomes in meiosis (Nokkala 1986).

The order Neuroptera belongs to the superorder Neuropterida, which comprises another two orders, namely, Raphidioptera with two extant families, Raphidiidae and Inocelliidae, and Megaloptera with two extant families, Corydalidae and Sialidae (Aspöck and Aspöck 2007). Interestingly, the Neuroptera share the “distance pairing” of sex chromosomes with Raphidioptera (Naville and de Beaumont 1936, Hughes-Schrader 1975a) but not with Megaloptera. In the latter group, all hitherto studied species, which belong to the single family Corydalidae, show another very specific “parachute-like” sex bivalent in spermatocyte meiosis (Hughes-Schrader 1980, Takeuchi et al. 2002). In this case, the X and Y chromosomes form a pseudo-bivalent that is situated together with the autosomes on the equator of the spindle and segregates synchronously with them at the first meiotic anaphase. This unique meiotic sex chromosome configuration called Xy_p_ (Smith 1950) is the well-known characteristic feature of the related order Coleoptera, and is encountered in almost all coleopteran families. Therefore Xy_p_ is considered ancestral for beetles (Smith 1950), at least for the suborder Polyphaga (Petitpierre 1987).

The variety and distribution of sex chromosome systems in different orders of the class Insecta have been comprehensively reviewed by Blackman (1995). The X(0) system was shown to predominate in the lower orders and is considered as ancestral condition for several major groups and for Insecta as a whole. The XY systems when occur are all derived from an X(0) one. The sex chromosome systems seem to provide useful phylogenetic evidence. Within Holometabola orders, besides simple X(0) and XY, there are some peculiar systems, e.g., those involving female heterogamety (XY/XX or ZW/ZZ) shared by Lepidoptera and Trichoptera, haplodiploid sex determination characteristic of Hymenoptera, and some others. Of these, distance pairing of the X and Y chromosomes in spermatocyte meiosis and the parachute Xy_p_ system are hypothesized to be synapomorphies respectively of the clade Neuroptera + Raphidioptera and of the clade Megaloptera + Coleoptera (Blackman 1995, Takeuchi et al. 2002).

## References

[B1] Angus RB Clery MJ Carter JC Wenczek DE (2013) Karyotypes of some medium-sized Dytiscidae (Agabinae and Colymbetinae) (Coleoptera). Comparative Cytogenetics 7: 171–190. doi: 10.3897/CompCytogen.v7i2.5223 24260699 10.3897/CompCytogen.v7i2.5223 PMC3833752

[B2] Asana JJ Kichijo H (1936) The chromosomes of six species of antlions (Neuroptera). Journal of the Faculty of Science, Hokkaido University (VI) 5: 121–136.

[B3] Aspöck U (2002) Phylogeny of the Neuropterida (Insecta: Holometabola). Zoologica Scripta 31: 51–55. doi: 10.1046/j.0300-3256.2001.00087.x

[B4] Aspöck U Aspöck H (2007) Verbliebene Vielfalt vergangener Blüte. Zur Evolution, Phylogenie und Biodiversität der Neuropterida (Insecta: Endopterygota). Denisia 20: 451–516.

[B5] Aspöck U Haring H Aspöck H (2012) The phylogeny of the Neuropterida: long lasting and current controversies and challenges (Insecta: Endopterygota). Arthropod Systematics & Phylogeny 70: 119–129.

[B6] Banks N (1899) A classification of the North American Myrmeleonidae. Canadian Entomologist 31: 67–71. doi: 10.4039/Ent3167-3

[B7] Banks N (1927) Revision of the Nearctic Myrmeleonidae. Bulletin of the Museum of Comparative Zoology at Harvard College 68: 1–84.

[B8] Blackman RL (1995) Sex determination in insects. Insect Reproduction. In: Leather SR Hardie J (Eds) CRC Press, Boca Raton, Florida, USA, 57–97.

[B9] Blackmon H Demuth J (2014) Coleoptera Karyotype Database. https://www.uta.edu/karyodb/ [last updated 19 December 2014]

[B10] Blackmon H Demuth J (2015) Coleoptera Karyotype Database. The Coleopterists Bulletin 69(1): 174–175. doi: 10.1649/0010-065X-69.1.174

[B11] Fischer K Hölzel H Kral K (2006) Divided and undivided compound eyes in Ascalaphidae (Insecta, Neuroptera) and their functional and phylogenetic significance. Journal of Zoological Systematics and Evolutionary Research 44: 285–289. doi: 10.1111/j.1439-0469.2006.00373.x

[B12] Gavrilov IA (2007) A catalogue of chromosome numbers and genetic systems of scale insects (Homoptera: Coccinea) of the world. Israel Journal of Entomology 37: 1–45.

[B13] Gavrilov-Zimin IA Stekolshchikov AV Gautam DC (2015) General trends of chromosomal evolution in Aphidococca (Insecta, Homoptera, Aphidinea+Coccinea). Comparative Cytogenetics 9: 335–422. doi: 10.3897/CompCytogen.v9i3.4930 26312130 10.3897/CompCytogen.v9i3.4930 PMC4547034

[B14] Gokhman VE (2009) Karyotypes of Parasitic Hymenoptera. Springer Science+Business Media B.V., Dordrecht, XIII + 183 pp. doi: 10.1007/978-1-4020-9807-9

[B15] Grozeva S Nokkala S (1996) Chromosomes and their meiotic behavior in two families of the primitive infraorder Dipsocoromorpha (Heteroptera). Hereditas 125: 31–36. doi: 10.1111/j.1601-5223.1996.t01-1-00031.x

[B16] Haring E Aspöck U (2004) Phylogeny of the Neuropterida: a first molecular approach. Systematic Entomology 29: 415–430. doi: 10.1111/j.0307-6970.2004.00263.x

[B17] Hirai H (1955a) Chromosome studies in the Neuroptera III. Chromosomes of seven species of the Myrmeleonoidea. Zoological Magazine 64: 370–374. [In Japanese with English summary]

[B18] Hirai H (1955b) Cyto-taxonomical Studies in the Japanese Neuroptera III. The Myrmeleonoidea. Miscellaneous Reports of the Yamashina’s Institute for Ornithology and Zoology 7: 27–29. doi: 10.3312/jyio1952.1.297

[B19] Hölzel H (1976) Revision der europäischen Creoleon-Arten (Planipennia, Myrmeleonidae). Zeitschrift der Arbeitsgemeinschaft Österreichischer Entomologen 28: 33–38.

[B20] Hughes-Schrader S (1969) Distance segregation and compound sex chromosomes in mantispids (Neuroptera: Mantispidae). Chromosoma 27: 109–129. doi: 10.1007/BF00326139 5364937 10.1007/BF00326139

[B21] Hughes-Schrader S (1975a) Male meiosis in camel-flies (Raphidioptera: Neuropteroidea). Chromosoma 51: 99–110. doi: 10.1007/BF00319828 1140044 10.1007/BF00319828

[B22] Hughes-Schrader S (1975b) Segregational mechanisms of sex chromosomes in spongillaflies (Neuroptera: Sisyridae). Chromosoma 52: 1–10. doi: 10.1007/BF00285784 1175454 10.1007/BF00285784

[B23] Hughes-Schrader S (1979) Diversity of chromosomal segregational mechanisms in mantispids (Neuroptera: Mantispidae). Chromosoma 75: 1–17. doi: 10.1007/BF00330620

[B24] Hughes-Schrader S (1980) Segregational mechanisms of sex chromosomes of Megaloptera (Neuropteroidea). Chromosoma 81: 307–314. doi: 10.1007/BF00368144 10.1007/BF00285784 1175454

[B25] Hughes-Schrader S (1983) Chromosomal segregational mechanisms in ant-lions (Myrmeleontidae, Neuroptera). Chromosoma 88: 256–264. doi: 10.1007/BF00292902

[B26] Ikeda K Kichijo H (1935) On the chromosomes of two species of Myrmeleonidae. Zoological Magazine (Tokyo) 47: 790–793. [In Japanese]

[B27] Katayama H (1939) On the chromosomes of *Hybris subjacens* Walk. (Neuroptera: Ascalaphidae). Japanese Journal of Genetics 15: 75–77. doi: 10.1266/jjg.15.75 [In Japanese with English summary]

[B28] King M (1993) Species Evolution: The Role of Chromosome Change. Cambridge University Press, New York, xxi + 336 pp.

[B29] Klok CJ Chown SL (1993) Karyotypes of five species of southern African Myrmeleontidae (Neuroptera). African Entomology 1: 29–33.

[B30] Krivokhatsky VA (2011) Antlions (Neuroptera: Myrmeleontidae) of Russia. KMK Scientific Press Ltd, St. Petersburg – Moscow, 334 pp [In Russian]

[B31] Kuznetsova V Aguin-Pombo D (2015) Comparative cytogenetics of Auchenorrhyncha (Hemiptera, Homoptera): a review. In: Lukhtanov VA Kuznetsova VG Grozeva S Golub NV (Eds) Genetic and cytogenetic structure of biological diversity in insects. ZooKeys 538: 63–93. doi: 10.3897/zookeys.538.6724 10.3897/zookeys.538.6724 PMC4722918 26807037

[B32] Kuznetsova VG Grozeva S Nokkala S Nokkala C (2011) Cytogenetics of the true bug infraorder Cimicomorpha (Hemiptera: Heteroptera): a review. ZooKeys 154: 31–70. doi: 10.3897/zookeys.154.1953 22287915 10.3897/zookeys.154.1953 PMC3238039

[B33] Lukhtanov VA (2014) Chromosome number evolution in skippers (Lepidoptera, Hesperiidae). Comparative Cytogenetics 8: 275–291. doi: 10.3897/CompCytogen.v8i4.8789 25610542 10.3897/CompCytogen.v8i4.8789 PMC4296715

[B34] Mansell MW (1999) Evolution and success of antlions (Neuropterida: Neuroptera, Myrmeleontidae). Stapfia 60: 49–58.

[B35] Naville A De Beaumont J (1932) Les chromosomes de quelques espèces de Névroptères. Compte rendu de séances de la Société de physique et d’histoire naturelle de Genève 49: 156–158.

[B36] Naville A De Beaumont J (1933) Recherches sur les chromosomes des Névroptères. Archives d’Anatomie Microscopique 29: 199–243.

[B37] Naville A De Beaumont J (1936) Recherches sur les chromosomes des Névroptères. Archives d’Anatomie Microscopique 32: 271–302.

[B38] New TR (1985a) A revision of the Australian Myrmeleontidae (Insecta: Neuroptera). I. Introduction, Myrmeleontini, Protoplectrini. Australian Journal of Zoology, Supplementary Series 104: 1–90.

[B39] New TR (1985b) A revision of the Australian Myrmeleontidae (Insecta: Neuroptera). II. Dendroleontini. Australian Journal of Zoology, Supplementary Series 105: 1–170.

[B40] New TR (1985c) A revision of the Australian Myrmeleontidae (Insecta: Neuroptera). III. Distoleontini and Acanthaclisinae. Australian Journal of Zoology, Supplementary Series 106: 1–159.

[B41] Nokkala S (1986) The nonsignificance of distance pairing for the regular segregation of the sex chromosomes in *Hemerobius marginatus* male (Hemerobiidae, Neuroptera). Hereditas 105: 135–139. doi: 10.1111/j.1601-5223.1986.tb00650.x

[B42] Oguma K Asana J (1932) Additional data on the dragonfly chromosome, with a note on occurrence of X-Y chromosome in the ant-lion (Neuroptera). Journal of the Faculty of Science, Hokkaido University 6: 133–142.

[B43] Papeschi A Bressa MJ (2006) Evolutionary cytogenetics in Heteroptera. Journal of Biological Research 5: 3–21.

[B44] Petitpierre E (1987) Why beetles have strikingly different rates of chromosomal evolution? Elytron 1: 25–32.

[B45] Sekimoto S Yoshisawa K (2007) Discovery of the genus *Suhpalacsa* Lefèbvre (Neuroptera: Ascalaphidae: Ascalaphinae) in Japan, with description of a new species. Entomological Science 10: 81–86. doi: 10.1111/j.1479-8298.2006.00201.x

[B46] Smith SG (1950) The cyto-taxonomy of Coleoptera. The Canadian Entomologist 82: 58–68. doi: 10.4039/Ent8258-3

[B47] Stange LA (1994) Reclassification of the New World antlion genera formerly included in the tribe Brachynemurini. Insecta Mundi 8: 67–119.

[B48] Stange LA (2004) A systematic catalog, bibliography and classification of the world antlions (Insecta: Neuroptera: Myrmeleontidae). Memoirs of the American Entomological Institute 74: 1–565.

[B49] Takeuchi Y Lizuka K Yamada T (2002) Chromosomes of the Japanese dobsonfly *Protohermes grandis* (Megaloptera: Corydalidae). Chromosome Science 6: 49–1.

[B50] van der Weele HW (1908) Ascalaphiden. Collections Zoologiques du Baron Edm. de Sélys Longchamps. Catalogue Systématique et Descriptif, Bruxelles, 8: 1–326.

[B51] Warchałowska-Śliwa E Heller KG Maryańska-Nadachowska A (2005) Cytogenetic variability of European Tettigoniinae (Orthoptera, Tettigoniidae): karyotypes, C- and Ag-NOR-banding. Folia biologica (Krakow) 53: 161–171. doi: 10.3409/173491605775142800 10.3409/173491605775142800 19058538

[B52] White MJD (1973) Animal cytogenetics and evolution. Cambridge University Press, Cambridge, 961 pp.

[B53] Winterton SL Hardy NB Wiegmann BM (2010) On wings of lace; phylogeny and Bayesian divergence time estimates of Neuropterida (Insecta) based on morphological and molecular data. Systematic Entomology 35: 349–378. doi: 10.1111/j.1365-3113.2010.00521.x

